# Can peer effects explain prescribing appropriateness? a social network analysis

**DOI:** 10.1186/s12874-023-02048-7

**Published:** 2023-10-28

**Authors:** Sophie Y. Wang, Nicolas Larrain, Oliver Groene

**Affiliations:** 1 Hamburg Center for Health Economics, Esplanade 36, 20354 Hamburg, Germany; 2 OptiMedis AG, Buchardstraße 17, 20095 Hamburg, Germany; 3 Institute of Health Policy, Management and Evaluation, University of Toronto, Toronto, Canada; 4 Employment, Labour and Social Affairs, Health Division, OECD, 2 Rue André Pascal, Cedex 16, 75775 Paris, France; 5 Faculty of Management, Economics and Society, University of Witten, Alfred-Herrhausen-Straße 50, 58455 HerdeckeWitten, Germany

**Keywords:** Social network analysis, Inappropriate prescribing, Physician networks, Potentially inappropriate medications, Peer effect

## Abstract

**Background:**

Optimizing prescribing practices is important due to the substantial clinical and financial costs of polypharmacy and an increasingly aging population. Prior research shows the importance of social relationships in driving prescribing behaviour. Using social network analysis, we examine the relationship between a physician practices’ connectedness to peers and their prescribing performance in two German regions.

**Methods:**

We first mapped physician practice networks using links established between two practices that share 8 or more patients; we calculated network-level (density, average path length) and node-level measures (degree, betweenness, eigenvector). We defined prescribing performance as the total number of inappropriate medications prescribed or appropriate medications not prescribed (PIMs) to senior patients (over the age of 65) during the calendar year 2016. We used FORTA (Fit fOR The Aged) algorithm to classify medication appropriateness. Negative binomial regression models estimate the association between node-level measures and prescribing performance of physician practices controlling for patient comorbidity, provider specialization, percentage of seniors in practice, and region. We conducted two sensitivity analyses to test the robustness of our findings – i) limiting the network mapping to patients younger than 65; ii) limiting the network ties to practices that share more than 25 patients.

**Results:**

We mapped two patient-sharing networks including 436 and 270 physician practices involving 28,508 and 20,935 patients and consisting of 217,126 and 154,274 claims in the two regions respectively. Regression analyses showed a practice’s network connectedness as represented by degree, betweenness, and eigenvector centrality, is significantly negatively associated with prescribing performance (degree—bottom vs. top quartile aRR = 0.04, 95%CI: 0.035,0.045; betweenness—bottom vs. top quartile aRR = 0.063 95%CI: 0.052,0.077; eigenvector—bottom vs. top quartile aRR = 0.039, 95%CI: 0.034,0.044).

**Conclusions:**

Our study provides evidence that physician practice prescribing performance is associated with their peer connections and position within their network. We conclude that practices occupying strategic positions at the edge of networks with advantageous access to novel information are associated with better prescribing outcomes, whereas highly connected practices embedded in insulated information environments are associated with poor prescribing performance.

**Supplementary Information:**

The online version contains supplementary material available at 10.1186/s12874-023-02048-7.

## Introduction

Optimizing prescribing practices is a priority in healthcare due to the substantial clinical and financial costs of drug-related complications. This is particularly a problem among seniors: in Germany, seniors over the age of 65 comprise 20% of the total population, and a regional study estimated that 22% of seniors are prescribed at least one potentially inappropriate medication [1, 2]. In 2009, the direct cost of prescribing potentially inappropriate medications was estimated at €387.8 million in Germany, which does not include other downstream expenditures such as hospitalizations and emergency department visits [3].

Polypharmacy, generally defined as the concomitant and long-term use of five or more medications, is a direct consequence of inappropriate prescribing and is associated with increased hospitalization, mortality, and adverse drug events [4–6]. Seniors, typically living with multiple chronic conditions, are at increased risk of polypharmacy due to their multiple interactions with different specialists who may prescribe medications for different conditions without regular medication review [7]. This is further exacerbated by the proliferation of single disease-specific guidelines recommended for use to prescribers, which has been shown to lead to a number of adverse drug interactions [8]. Multiple groups have argued for an interprofessional and team-based approach to reduce polypharmacy, considering patient complexity and multimorbidity [9, 10]. There is a paucity of data, however, on how interprofessional teams collaborate to manage polypharmacy.

The Fit fOR The Aged (FORTA) classification was first developed in 2010 through consensus by German-based experts to support physicians in improving appropriate prescribing for seniors (aged over 65) in the German context [11]. This was created in response to the paucity of evidence-based guidelines for the senior patients combined with the high level of polypharmacy and potentially inappropriate medications (PIMs) observed [11]. FORTA scores provide an assessment of prescribing quality for a patient by cross-referencing prescribed medications with a patient’s diagnosis to detect over- and under-prescribing based on drug-drug interactions. Research has shown that application of FORTA in clinical workflow improved appropriate prescribing among prescribers for seniors (over the age of 65) in Germany [11, 12] and has a comparatively better performance in detecting potentially inappropriate medications (PIMs) [13]; it has subsequently been applied to different settings following expert consensus meetings [14–16].

Recent decades have seen the rise of both evidence-based prescribing—prescribing based on the best available, critically appraised evidence—and rational prescribing, wherein judicious prescribing maximizes effectiveness while minimizing harm and avoiding waste [17]. However, social relationships remain an important driver of prescribing decisions [18–21]: for example, research on diffusion of new medications has shown that prescribers are highly influenced by peer adoption [22] and pharmaceutical manufacturer exposure [23, 24]. Additionally, a growing body of literature in behavioural science demonstrates the applicability of socially-influenced interventions such as peer comparison to improve guideline-concordant practices among prescribers [25]. Influences on prescribing practice are not limited to physicians within the same practice [26, 27], but also among physicians that share patients with one another [28]. This finding may be particularly relevant to seniors who typically have comparatively higher rates of comorbidities; and as a result, require multiple prescribers to be aware of their medication history and coordinate treatment.

Such peer relationships can be studied using network analysis, which considers the interaction and dynamics between individuals within a system. Network analysis can reveal implicit relationships between different actors within a system, who act as communication channels for information exchange and social influence [29]. These relationship structures and the positioning of individual actors within a network has important implications on the behaviour, perceptions, and attitudes for the individual as well as those around them [29]. Network positioning of an individual is associated with performance measures in various sectors including education [30], engineering [31], and innovation [32]. Physician social networks have increasingly been the subject of study as increasing pressure is exerted on health systems to contain costs and improve quality [33].

Time constraints among healthcare providers and low survey response rates are significant barriers to network analysis in healthcare [33]. To overcome these barriers, Barnett and colleagues have used administrative claims data to identify connections between physicians based on shared patients, and showed that physicians who share patients are more likely to personally know each other, exchange information and informal advice [34–36]. Physician connectedness to others in their professional network is associated with improvements in patient outcomes including length of stay [37], mortality [38], and patient satisfaction [39]. This method has since been applied widely to characterize relationships among physicians in hospitals [40], and outpatient clinics [41], and at the organizational level in long term care facilities [42] and between hospitals [43].

Physician practices may share patients because of unresolved clinical problems that require complementary expertise, when different specialists are providing team-based care to a patient with complex and chronic disease, or when patients seek second opinions for further clinical information [44]. Apart from the latter patient-driven mechanisms for patient-sharing, the first two scenarios create a motivation for the physician involved to review the patient’s current and past medical history, and to either formally connect with a colleague that is also caring for the patient or to informally consult a colleague for management, ethical or therapeutic advice [45–47] as a form of information exchange. Additionally, physician relationships may be reinforced in professional practice settings outside of clinical environments where acquaintance is established through shared patients.

Here we identify a patient-sharing provider network using German regional healthcare claims data and characterize the association between a physician practice’s level of connectedness to peers and its performance in prescribing quality. We hypothesized that more connected practices are better positioned to exercise their social capital and draw on the knowledge of peers, which may guide them to adopt quality prescribing practice and exhibit better performance. By applying a method previously validated in large medical centers and community hospitals in the United States [34], we demonstrate the transferability to German data, and also to smaller geographic regions, where analytical implications are more relevant for local decision-making.

## Methods

Social networks can be used to reveal underlying relationships between different actors in a system. Studying how different actors are connected can lead to insights on the relative social capital individuals have to draw on and how others in the proximity of their network might influence them. Networks consist of nodes and ties that represent how nodes are connected to one another. In our study, each node represents a physician practice, and a tie represents the relationship between two physician practices, inferred using the number of shared patients between the two corresponding practices.

### Study design

This is a retrospective, observational study using administrative claims data to examine the relationship between a physician practice’s positioning within a social network and their prescribing performance for senior patients in 2016. We follow the research framework for network analysis using administrative claims data proposed by Uddin following a systematic review of this field of work [48]. We used claims data from two insurance companies in the respective geographic regions in Billstedt-Horn and Werra-Meissner Kreis, which covers roughly 45% and 23% of the patient population in the two regions respectively. The majority of existing work on mapping patient sharing networks using administrative data is based in the United States [33]. While recent research set in the United States estimated differences in network statistic when different sources of insurance claims data is used to map the network [49], we assume that the insuree demographic is similarly distributed between health insurance companies in Germany. This is because in the German healthcare system, individuals have free choice of the health insurance company (i.e., sickness fund), and risk adjustment schemes are in place to minimize incentives for adverse selection by the insurance companies. Thus, any differences between the two subsamples are attributed to regional differences. Practice claims data were obtained through established data-sharing agreements with the regional sickness fund. We used pseudonymized physician practice and patient data in this study and thus our study was exempt from institutional ethics review at the University of Hamburg.

### Research setting

This study is set in two areas in Germany: Billstedt-Horn, and Werra-Meißner-Kreis. Billstedt-Horn is a multicultural and urban district with a population of roughly 110,000 in Northern Germany [50]. Werra-Meißner-Kreis is a region in rural central Germany consisting of a little over 100,000 population and have a larger share of elderly population. In our study, we limit the analysis to ambulatory clinics, which include both primary care practices and specialist clinics working as solo or group practice.

### Study samples

**Fig. 1:**
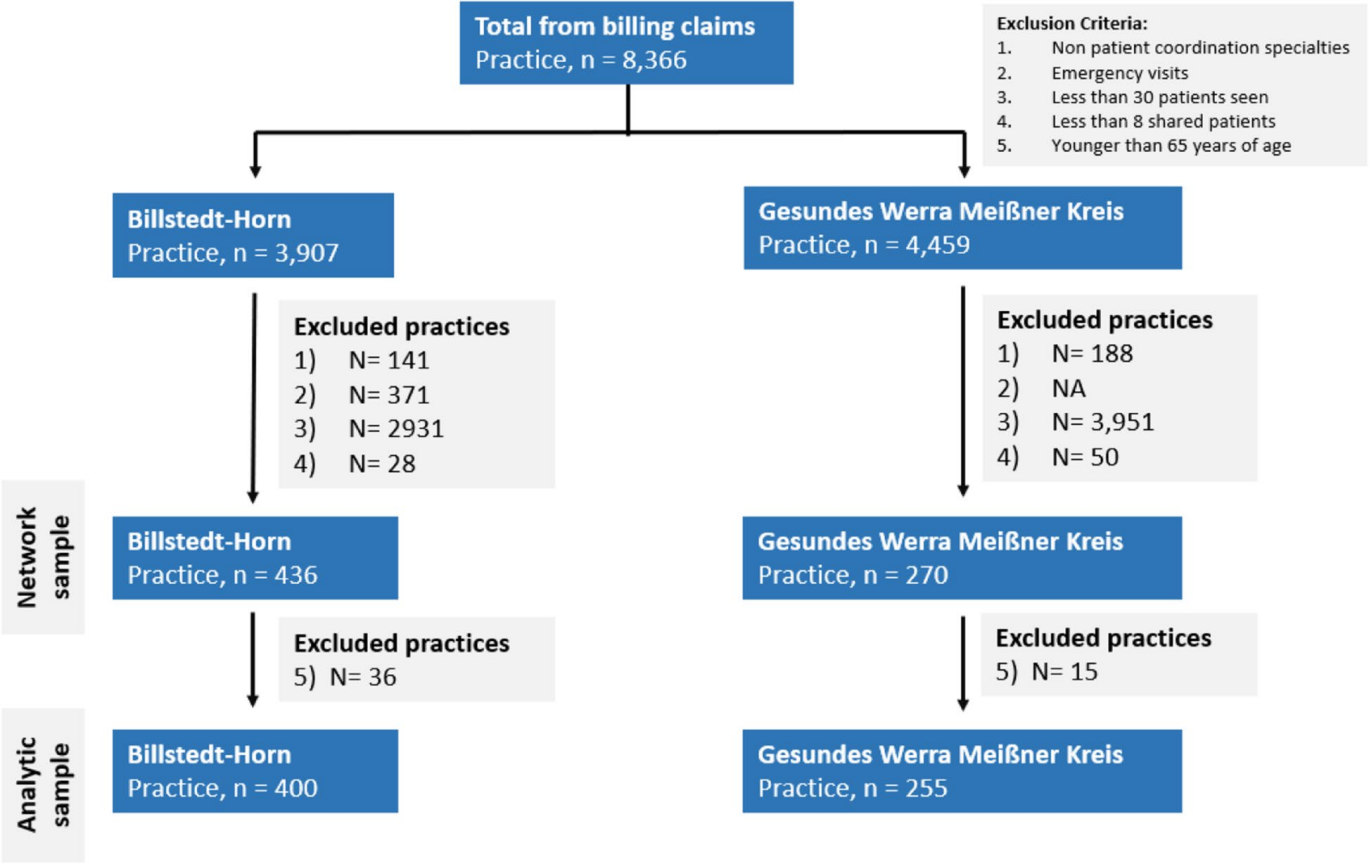
Sample size for network mapping and selection criteria

The population in this study included physician practices who were involved in the treatment and care of patients insured by the insurance companies in the year 2016 in Billstedt-Horn and Werra-Meissner-Kreis respectively. To identify eligible claims for network mapping, we started from the full claims record, and excluded practices whose specialty is not typically responsible for direct patient care and hence coordination (anesthesiology, radiology, pathology, radiotherapy, and nuclear medicine); claims that were categorized as emergency visits; and practices that had fewer than 30 patients in the 2016 calendar year. By filtering out practices with fewer than 30 unique patients that filed claims within the year, we effectively exclude practices that may not have a strong presence in the regional professional social network. As the claims data is based on the patient postal code, practices excluded may be physicians that patients seek out outside of the region of interest in this study; thus, while a large percentage of practices are excluded based on this criterion (Fig. 1), we do not believe that the resulting sample introduces selection bias.

To reach the final analytic sample, we further excluded practices that do not have claims records of patients over the age of 65 due to the outcome variable of interest in this study. The physician practice sample used to map the patient-sharing network and the analytic sample is shown in Fig. 1.

### Mapping patient-sharing network and analysis

We map patient-sharing networks between practices in the same geographic region using administrative claims data. This method of mapping affiliation networks [51] was previously validated [34] and has been performed on outpatient clinic data [41]. Affiliation networks show connections between actors based on common events or co-membership, assuming that this indicates an underlying (or potential) social tie [51]. Using this method of network mapping, connections between physician practices are based on linkages established through common patients shared between the two [26, 34, 51]. In this view, shared patients act as a conduit for diffusion of clinical practices between those that provide care.

Patient-sharing networks were mapped separately for the two regions included in this study using a previously validated method developed by Barnett and colleagues [34]. Accounting for practical considerations highlighted by Dugoff and colleagues in network identification [33], we applied the following criteria for inclusion of practices in our study: i) medical specialities whose scope of practice includes patient coordination with others; ii) non-emergency visits; iii) claims within the calendar year 2016; iv) more than 30 patients seen in 2016; v) include patients over the age of 65 years old. Specific details on steps taken to map the network can be found in the additional file (Supplement—S1). Each node within our network represents a physician practice, and an edge implies an information-sharing relationship between two practices based on more than 8 shared patients. Using this method of network mapping and in line with prior research, we infer an information-sharing relationship between two physicians that share a significant number of patients and that the extent to which they are connected is reflected by the number of patients shared.

### Network-level and node-level measures

We characterize the degree of network integration using network-level indicators and the structural position of a practice within the network using node-level centrality measures (Table 1). Network-level indicators include density and average path length. Density represents how densely connected the nodes are to all other nodes in each network; average path length captures the average of the shortest paths between any two nodes in the network.

**Table 1:** Definition of node-level and network-level measures

Measure	Represents	What does this mean?
**Node-level measures**		
Degree	Number of other practices connected to the practice of interest through patients shared	A measure of the quantity of potential contacts representing available resources to draw on. A high degree also represents a prominent role in information channel in the practice’s immediate network [52]
Betweenness	The total number of shortest paths between any two given practices within the network	Practices with high betweenness scores are well-positioned to receive information early [52], and can be interpreted as a measure of potential control given others’ dependence on the practice [53]
Eigenvector centrality	A high eigenvector score represents that a given practice is connected to other well-connected practices	A measure of influence over the longer-term (“a practice’s long-term equilibrium risk of receiving traffic is a function of the risk level of its contacts” [52]
**Network-level measures**		
Network density	The proportion of existing ties over all possible ties among nodes in network	Lower density = less connected network
Average path length	The average shortest paths for all pairs of nodes in network	A measure of local connectivity; shorter average path lengths indicate higher connectivity

Node-level indicators we used to characterize centrality include degree, betweenness, and eigenvector centrality [35]. These centrality measures were chosen based on our assumptions of how information flows in our shared-patient network [52] and informed by theory [54, 55]. Degree measures the number of other practices the practice of interest is directly connected to through shared patients with each practice weighted equally. This measure implicates the level of available intangible resources a practice may draw upon and represents a communication focal point for those to whom this node is connected. Betweenness measures the number of times a practice is located on the intermediary step of all shortest paths linking two other practices in the network. Graphically, practices with a high betweenness centrality will be positioned in the middle of a network rather than on the periphery. Practices with a high betweenness score can be interpreted as having great influence on their network peers and having greater access to the information flow among practices within the network. Eigenvector centrality is a measure of the practice’s influence on the overall practice network over the long term, whereby a high eigenvector value indicates that the practice of interest is linked to other practices that are themselves highly connected.

### Study variables

#### Outcome prescribing quality measure

To assess practice prescribing quality for senior patients, we used the FORTA tool that classifies prescriptions as a PIM using an evidence-based algorithm [11]. The FORTA tool provides a numeric score of PIMs received for each patient (over the age of 65) by cross-referencing the patient’s diagnosis, history, and medication profile; this score may reflect repeating PIMs receives over multiple clinical encounters if left unresolved. A high FORTA score for a patient indicates a high number of PIMs prescribed, including both over- and under- prescribing, which may be attributed to all physicians who were responsible for the patient.

As our unit of analysis was at the practice level, the prescribing quality measure is aggregated at the practice level. Specifically, the practice prescribing quality is calculated by summing the cumulative annual FORTA score of each patient in the practice. An increase in one summed FORTA score represents an increase in one PIM prescribed to a patient under the care of the practice during the calendar year. A high summed FORTA score represents poor prescribing quality among the physician practice; and a low summed FORTA score represents good prescribing quality.

#### Predictors – physician related

Apart from centrality measures as described above, we considered supply-side factors identified in literature that affect prescribing quality. These include practice patient load [56], physician age [56], rural/urban location [57], payment and incentive schemes [58, 59], physician specialization, and physician education [60, 61]. The claims data to which we had access allowed us to control for the percentage of seniors in each practice, the practice specialty, and the practice region. We controlled for the percentage of seniors in each practice as senior patients tend to have more co-morbidities, and thus to have more concurrent medications and opportunities for prescription error. Though it is conceivable that the total number of patients under the care of one practice may affect the prescribing quality, due to its high correlation with the percentage of seniors variable, it was not included in model as a control. Practice specialty was deduced based on service practices predominantly filed for claims within the calendar year of 2016. Primary care providers indicate practices that are categorized as “Hausarzt” or general practice medicine and gynecologists who serve as the first point of contact for most patients and are expected to have more of a role in medication coordination. Additionally, due to the broad scope of practice of general practitioners, specialists’ knowledge of appropriate medications are generally deferred to [62]. We also controlled for the region, where Billstedt Horn represented an urban and moderately deprived region while Werra Meissner Kreis represents a rural and monoethnic community.

#### Predictors – patient related

We used the Charlson comorbidity index to control for patient comorbidity, as patients with more comorbidities are likely to be prescribed more medications, which may inadvertently drive-up medication errors. Because it would be impractical to include all comorbidity variables in a regression model due to issues of multicollinearity and inadequate degrees of freedom, we chose to use a composite measure. The Charlson comorbidity index considers the severity of specific diseases and the patient’s age in the weighted score; it is widely used as a mechanism to control for patient comorbidity when analyzing administrative data [63]. We transform the patient-level Charlson comorbidity score to a physician practice-level variable by averaging the Charlson comorbidity score of all patients in the practice.

### Statistical analysis

Following the mapping of physician practice network, we conducted descriptive analysis in both regions of interest including patient demographics, provider coverage, and network parameters; we also explored the distribution of outcome variable in both the healthcare regions. We then conducted bivariate analyses using Spearman’s rank correlation to describe the strength of association and direction between the centrality measures (degree, betweenness, and eigenvector) and potentially inappropriate prescriptions (summed FORTA score). Spearman’s rank correlation was used as it does not make assumptions regarding the underlying distribution of the data, which did not follow a normal distribution. Variables were assessed for potential multicollinearity by measuring the variance inflation factors (VIF) and balanced with omitted variable bias when constructing the regression model.

We defined quartiles within each region for all network centrality measures (degree, betweenness, and eigenvector) and treated the quartiles as categorical variables. Data from both regions were pooled in the regression analyses to increase the power to determine the effect. Each region was identified using a dummy variable to account for underlying differences. We used a negative binomial regression to estimate the adjusted incidence rate ratio of prescribing a potentially inappropriate prescription due to the overdispersed count outcome variable. It is important to note that the unit of analysis is at the practice level, and thus patient-associated scores were aggregated.

Our model specification is as follows:

$$\mathrm{y}=\mathrm{\alpha }+ {\upbeta }_{1}\mathrm{\, centrality}+ {\upbeta }_{2}\mathrm{\, percentage\, of\, seniors}+ {\upbeta }_{3}\mathrm{ \,mean \,Charlson \,score}+ {\upbeta }_{4 }\mathrm{\,Primary\, Care \,Provider}+ {\upbeta }_{5}\mathrm{\, region}+\upvarepsilon$$

where: centrality measure represents degree, closeness, or eigenvector centrality (Table 1); percentage of seniors refer to the percentage of elderly patients (over the age of 65) over the total practice patient panel size; mean Charlson score represents the mean Charlson comorbidity score of all included patients (seniors) belonging to the practice; primary care provider is a dummy variable, where practices specialize in either general internal medicine, family practice, or gynecology were assigned a value of 1, and 0 otherwise; finally, region is also a dummy variable that identifies if the practice is in Billstedt-Horn or Werra-Meissner-Kreis and is used as a proxy for rural or urban context.

#### Sensitivity analysis

We conducted two separate sensitivity analyses to check for robustness of effects in different subsamples. First, we address the challenge of endogeneity between the network measures and the outcome measure by remapping the social network with only physician practices that shared younger patients (defined as under the age of 65) and deriving practice-level centrality measures. We then retained our original analytical sample (from main analysis) and updated the associated centrality measures derived from physician practices sharing only younger patients. By doing so, we isolate the effects of practice centrality on prescribing quality from the contribution of senior patients who are more likely to have multiple comorbidities and thus simultaneously increasing the number of physician visits (contributing to a higher centrality measure) and increasing the FORTA scores. Second, we tested the stability of effects by remapping the network using an increased threshold of 25 shared patients. By increasing the threshold of shared patients in which ties between practices are drawn, we effectively increase the specificity of linkages (i.e., decreased likelihood of drawling linkages between spurious connections) [34]. In other words, we increase the certainty that linkages between practices detected represent true connections.

All statistical analyses were performed using the R statistical software (version 4.0.3).

## Results

The two patient-sharing networks we mapped—Billstedt-Horn and Werra Meissner Kreis—included 436 and 270 practices; 28,508 and 20,935 patients; and consisted of 217,126 and 154,274 claims in the 2016 calendar year, respectively. We further excluded patients under the age of 65 and practices that did not care for patients over the age of 65 to reach the analytical sample consisting in total of 659 practices (Billstedt-Horn: 401; Werra Meissner Kreis: 269) (Fig. 1). Within the analytical sample, the patient population in both regions have similar sex distribution; though in Billstedt-Horn, patients are slightly older (median of 75 years of age, compared to 71 in Werra Meissner Kreis) and have a higher comorbidity score (Charlson index of 4.25, compared to 4 in Werra Meissner Kreis) (Table 2). Physician practices have larger patients under their care in Werra Meissner Kreis (median of 128 patients, IQR = 382), as compared to Billstedt-Horn (median of 95 patients, IQR = 203); and Billstedt-Horn have a larger percentage of primary care providers among all practices (Billstedt-Horn 38%; Werra Meissner Kreis 30%). In the two networks included, Werra Meissner Kreis appears to be a more connected network with a higher density (0.16, compared to 0.08 in Billstedt-Horn) and shorter mean distance (1.99, compared to 2.10 in Billstedt-Horn). Both networks have a longer mean distance compared to the average generated over 1000 random graphs with the same number of nodes, which indicates a less connected network compared to what would be expected of an average network with the same number of nodes. While the Billstedt-Horn network had a slightly higher percentage of same-specialty tie (BH: 6.76%; WMK: 4.40%), the proportion that are primary care provider is similarly high across both regions (BH: 82%; WMK: 84%) (Table 3).

**Table 2:** Characteristics of patients and physicians by geographical area in analytical sample

	**Billstedt-Horn**	**Werra Meissner Kreis**
**Patient characteristics**		
Number of unique patients	5287	2870
Female, n (%)	2872 (54)	1501 (52)
Age, median (IQR)	75 (12)	71 (11)
Charlson score, median (IQR)	4.25 (3)	4.0 (2.25)
**Practice characteristics**		
Number of unique practices	401	258
Number of patients, median (IQR)	95 (203)	128 (382)
Primary Care Provider^a^, n (%)	153 (38)	77 (30)
**Node centrality**		
Degree, median (IQR)	14 (43)	27 (64)
Betweenness, median (IQR)	104 (278)	79 (181)
Eigenvector, median (IQR)	0.012 (0.05)	0.039 (0.15)

^a^Primary care provider defined as general practitioner or gynecologist

**Table 3:** Network level measures by geographical region

	**Billstedt-Horn**	**Werra Meissner Kreis**
Density	0.08	0.16
Mean distance	2.10	1.99
Percentage of same-specialty ties	6.76%	4.40%
Percentage of same-specialty ties that are primary care	82%	84%

Results from the negative binomial regression are presented in Table 4, where degree, betweenness, and eigenvector centrality are shown separately. For each centrality measure, we present three different models: model 1 contains only the centrality measure; model 2 is a control-only model; and model 3 is the full model with control variables included. For ease of interpretation, we also present results as adjusted incidence rate ratio (IRR) for centrality measures and number of potentially inappropriate prescriptions (Table 5). Our results showed that network centrality measures were significantly associated with PIMs. We found a significant negative relationship between the centrality measures degree, betweenness, and eigenvector and prescribing a PIM (Table 5). Those in the bottom quartile of degree centrality had an incidence rate 0.4 times that of practices in the top quartile (IRR = 0.040, 95%CI (0.035, 0.045)); those in the bottom quartile of betweenness centrality had an incidence rate 0.06 times that of practices in the top quartile (IRR = 0.063, 95%CI (0.052, 0.077)); and those in the bottom quartile of eigenvector centrality had an incidence rate 0.04 times that of practices in the top quartile (IRR = 0.039, 95%CI (0.034, 0.044)).

**Table 4:** Negative binomial regressions estimating practice level prescribing quality with practice-level centrality measures defined as quartiles: (a) degree centrality; (b) betweenness centrality; (c) eigenvector centrality

(a) Decree centrality in quartiles			
	Model 1	Model 2	Model 3
(Intercept)	85 ***	4.83***	6.68***
	(0.064)	(0.25)	(0.13)
Degree (Ref: Q1)			
Q2	-15.21 ***		-1.56***
	(-16.9)		(0.066)
Q3	-27.52***		-2.62***
	(0.090)		(0.067)
Q4	-35.01***		-3.23***
	(0.090)		(0.067)
Mean Charlson		0.11*	0.10***
		(0.049)	(0.024)
Percentage senior (> 65)		8.21***	5.09***
		(0.40)	(0.20)
Primary care provider (Ref: Specialist)		0.15 (0.11)	0.082 (0.052)
Region (Ref: Billstedt-Horn)		0.36 ***	0.24***
		(0.10)	(0.051)
N	638	626	640
AIC	9875	10,553	9410
(b) Betweenness centrality in quartiles			
	Model 1	Model 2	Model 3
(Intercept)	8.34***	4.83***	6.41***
	(0.084)	(0.25)	(0.19)
Betweenness: (Ref: Q1)			
Q2	-1.09***		-1.19***
	(0.12)		(0.096)
Q3	-1.68***		-1.88***
	(0.12)		(0.097)
Q4	-3.04***		-2.76***
	(0.12))		(0.097)
Mean Charlson		0.11*	0.021
		(0.049)	(0.035)
Percentage senior (> 65)		8.21***	6.98***
		(0.40)	(0.29)
Primary care provider (Ref: Specialist)		0.15 (0.11)	0.29*** (0.076)
Region (Ref: Billstedt-Horn)		0.36 ***	0.38***
		(0.10)	(0.074)
N	638	626	640
AIC	10,333	10,553	9988
(c) Eigenvector Centrality in quartiles			
	Model 1	Model 2	Model 3
(Intercept)	85 ***	4.83***	6.90***
	(0.064)	(0.25)	(0.13)
Eigenvector (Ref: Q1)			
Q2	-16.24 ***		-1.65***
	(0.087)		(0.065)
Q3	-27.54***		-2.61***
	(0.087)		(0.066)
Q4	-35.57***		-3.25***
	(0.088)		(0.067)
Mean Charlson		0.11*	0.084***
		(0.049)	(0.024)
Percentage senior (> 65)		8.21***	4.70***
		(0.40)	(0.20)
Primary care provider (Ref: Specialist)		0.15 (0.11)	-0.0079 (0.051)
Region (Ref: Billstedt-Horn)		0.36 ***	0.19***
		(0.10)	(0.050)
N	638	626	640
AIC	9829	10,553	9391

Standard errors are heteroskedasticity robust

^***^p < 0.001

^**^p < 0.01

^*^p < 0.05

**Table 5:** Association between potentially inappropriate prescriptions (summed FORTA scores) and network centrality measures in quartile in 2016. Results from negative binomial regression

	Degree		Betweenness		Eigenvector	
	IRR	95% CI	IRR	95% CI	IRR	95% CI
Centrality						
Q1 (ref)	1.00		1.00		1.00	
Q2	0.21	(0.19, 0.24)	0.31	(0.25, 0.37)	0.19	(0.17, 0.22)
Q3	0.073	(0.064, 0.083)	0.15	(0.13, 0.18)	0.074	(0.065, 0.084)
Q4	0.040	(0.035, 0.045)	0.063	(0.052, 0.077)	0.039	(0.034, 0.044)
Mean Charlson score of practice	1.11	(1.04, 1.17)	1.02	(0.94, 1.11)	1.09	(1.03, 1.15)
Percentage of senior patients	163	(100, 267)	1078	(508, 2317)	110	(68, 179)
Primary Care Provider (Ref: Specialist)	1.09	(0.98, 1.20)	1.34	(1.16, 1.56)	0.99	(0.90, 1.10)
Region (Ref: Billstedt-Horn)	1.27	(1.15, 1.40)	1.47	(1.26, 1.70)	1.20	(1.09, 1.33)

Each negative binomial regression model also includes mean Charlson core of practice, percentage of senior patients, primary care provider dummy variable, and region dummy variable as controls and uses the summed FORTA score as the response variable

*QI* Quartile 1 (most well-connected), *Q2* Quartile 2, *Q3*  Quartile 3, *Q4* Quartile 4, *IRR* Incidence rate ratio, *CI* Confidence Interval

Across the specified models, adjusted results show that many practice-level variables were consistently associated with PIMs (Table 4). We found that increases in mean Charlson score to be significantly associated with increased PIMs across all centrality measures. We found percentage of senior patients to have a significant positive association with PIMs across all centrality measures. We did not find a consistent association between primary care provider and PIM, however among models encompassing betweenness centrality, we observed a significant positive relationship. Lastly, we observed a consistent relationship between region and PIMs with Werra-Meissner-Kreis to have a significantly higher PIM as compared to Billstedt Horn.

We examined the consistency of the association between centrality measures and PIM by remapping the network with two alternative methods: i) including only clinical encounters involving younger patients (less than 65 years old); ii) increasing the threshold of shared patients that defines a tie between two practices from 8 to 25 patients. In both sensitivity analyses conducted, the results were in line with main analysis with slight changes in effect size, same directionality of association and significance (Table 6). From the first sensitivity analysis, we found that as degree of connectedness decreases, the incidence of a PIM also decreases (network including clinical encounter with young patients: adjusted IRR for practices in the bottom quartile had an incidence rate of 0.044 times that of practices in the top quartile (95%CI: 0.039, 0.050); network including only ties established by more than 25 shared patients: adjusted IRR for practices in the bottom quartile had an incidence rate of 0.059 times that of practices in the top quartile (95%CI: 0.050, 0.069)) (Table 6). We also found a similar negative association between PIM and betweenness centrality as well as eigenvector centrality (Table 6).Similar results were found for the second sensitivity analysis, where a positive association was observed between centrality measures degree, betweenness, and eigenvector centrality of practices and PIMs (Table 6). We found that results from both sensitivity analyses confirm the robustness of our findings. The models for all three centrality measures resulted in slight changes in effect size, but the same directionality and level of significance. Results from both sensitivity analyses confirm the robustness of our findings.

**Table 6:** Association between potentially inappropriate prescriptions (summed FORTA scores) and network centrality measures in quartile in 2016: (a) Regression models with centrality measures derived from networks mapped including only patients younger than 65 years of age (Billstedt-Horn: *N* = 413; Werra-Meissner-Kreis: *N* = 262); (b) Regression models with centrality measures derived from networks mapped including only ties consisting of more than 25 shared patients (Billstedt-Horn: *N* = 258; Werra-Meissner-Kreis: *N* = *194)*

(a)						
Centrality Measure	Degree		Betweenness		Eigenvector	
	IRR	95% CI	IRR	95% CI	IRR	95% CI
Q1 (ref)	1.00		1.00		1.00	
Q2	0.21	(0.18, 0.23)	0.35	(0.29, 0.43)	0.20	(0.18, 0.23)
Q3	0.078	(0.068, 0.089)	0.16	(0.13, 0.19)	0.077	(0.067, 0.088)
Q4	0.044	(0.039, 0.050)	0.059	(0.049, 0.072)	0.047	(0.041, 0.053)
(b)						
Centrality Measure	Degree		Betweenness		Eigenvector	
	IRR	95% CI	IRR	95% CI	IRR	95% CI
Q1 (ref)	1.00		1.00		1.00	
Q2	0.26	(0.23, 0.31)	0.48	(0.39, 0.59)	0.25	(0.22, 0.29)
Q3	0.11	(0.096, 0.13)	0.20	(0.16, 0.25)	0.12	(0.100, 0.134)
Q4	0.059	(0.050, 0.069)	0.076	(0.062, 0.095)	0.057	(0.049, 0.067)

## Discussion

Our research examines the structure and composition of shared-patient networks mapped between practices in two German healthcare areas using insurance claims data. We find that practice centrality is associated with prescribing quality for elderly patients when controlling for confounding factors; contrary to our hypothesis, the association is in the negative direction (i.e., more well-connected practices are associated with poorer prescribing quality). Research mapping social networks using administrative data has increased steadily in recent decades; while most studies are set in the US and Australia [33], studies in France [64] and Germany [65, 66] has been published in the last couple years. To our knowledge, this research is the first to apply this novel method to study prescribing performance for elderly patients in the German health system context. Prior research mainly focused on organizational, physician, and patient determinants of prescribing quality [67]. This study elucidates the influence of information-sharing relationships and the structural position within one’s network on prescribing quality outcome. By applying a replicable method leveraging administrative data, our research provides an additional tool for regional change management in designing quality improvement strategies.

In this study, we find practices that are well-connected to others (high degree centrality), well-positioned to broker information between network practices (high betweenness centrality) and connected to well-connected others (high eigenvector centrality), were more likely to be associated with an overall increased over- and under- prescribing scores (i.e., to have worse prescribing quality). We interpret this finding by expanding on the following: i) occupying positions on the network fringes may confer advantage in accessing novel information; ii) highly connected practices may be subject to an insulated information environment; iii) negative resource exchanges in professional networks.

This work draws upon social network theory, which posits that the embeddedness of each individual’s positioning within a network, and their connectedness to others, confer both advantages and drawbacks [68]. In turn, social capital theory can provide a foundation for describing and characterizing the observed results. Social capital, as first defined by Boudieu [69] and expanded upon by Burt [70], Coleman [71], and others, is the ability of actors to secure benefits such as information through membership in social networks or other structures [72]. Within the context of network structures, social capital theory can be conceived as two mutually exclusive yet reinforcing mechanisms—structural holes and social cohesion [73].

Access and exchange of knowledge and information is particularly salient in our study of prescribing quality. While clinical knowledge is typically imparted through textbooks, clinical guidelines and journal articles [19], research shows that physicians also learn directly through peer interactions [45–47] and may be influenced by their social milieu, particularly under high levels of uncertainty [19–21]. Following Burt’s structural hole theory, practices that are less connected in their local networks may act as a bridge to distinct communities, and may have a strategic advantage in accessing diverse information [73, 74]. Conversely, well-connected practices embedded in dense networks may be exposed to redundant information from peers that possess similar knowledge and information. This low information diversity among more central practices may be a barrier to integration of prescribing guidelines or novel clinical information and thereby reflect the higher over- and under-prescribing associated with practices that were highly connected. Others have shown that physicians reporting higher adoption of evidence-based medicine adoption were negatively associated with indicators of network prominence and centrality [75].

The social cohesion perspective proposes that individuals in highly connected networks are likely to demonstrate homogeneous behavior due to the spread of norms, ideas, and practices through social relationships [76, 77]. This phenomenon is further evidenced by the strong and restrictive social control that exists in knowledge-intensive networks [31]. Physicians conforming to local norms, etiquette, and hierarchical structure, even in instances where prescribing decisions should be challenged, is well documented in literature [18, 21]. One manifestation of strong social cohesion is the process of “groupthink” where a premature consensus is achieved based on group judgement, which may reduce the rationality of the decision or behaviour of a group [78]. Closely linked is the concept of “echo” whereby individuals access information through their peers and hence rely on the peers’ filtering and interpretation of information according to their prior knowledge and opinions [79]. Through socialization processes such as echo and groupthink, highly connected physician practices may develop prescribing patterns that depart from best evidence yet are locally endorsed and reinforced.

While a variety of benefits may be derived from social capital, negative exchange relations also exist and have been associated with individual performance [80] and described in healthcare teams as “hindrance networks” [81]. Hindrance networks can be characterized as the exchange of negative resources that may inhibit or hinder the individual or team’s performance, particularly when performance of individuals depends on access to information from others [81]. In a recent study of polypharmacy in Germany, general practitioners expressed concern regarding the infrequent and poor communication of medication changes during care transitions and following specialist appointments [82]. In the context of this study, prescribing quality among well-connected practices may be compromised due to differences in communication channels between prescribers in different settings, and the lack of structures to share patient medication history.

Poor prescribing performance associated with well-connected practices found in our study reflects a fragmented healthcare system where physicians who are positioned to provide coordinated care lack formal structures and incentives to support this practice. In Germany, the lack of electronic patient health records that service providers from different settings can access undermines their ability to provide coordination of care and medication reconciliation [6, 83]. Integrated care initiatives have been introduced in both regions under study in recent years. The focus on optimizing appropriate prescribing practices through integration of the FORTA algorithm in providers’ clinical workflow provides feedback on prescribing quality. Future research may well utilize this analysis to evaluate whether introduction of feedback mechanisms and care management programs improve care coordination within established professional networks.

### Implications for practice and policy

Our research generates several implications for practice and policy. As health systems restructure to improve care coordination for the patient, it is critical for system administrators and policy makers to recognize the importance of relationships and positioning within existing networks in shaping behaviour and performance.

Adherence to local prescribing norms creates a closed network where novel information, such as revised prescribing guidelines, fail to reach prescribers. Our findings that well-connected practices are associated with worse prescribing practices may be a result of practices conforming to local prescribing norms that depart from best practice guidelines. Implementing quality circles [84], where physician colleagues gather to discuss locally relevant quality improvement initiatives and exchange ideas, may present an opportunity to introduce information in an insular environment. Poss-Doering and colleagues found regular peer exchanges through quality circles was highly valued by physicians in primary care networks and contributed to improved guideline-concordant antibiotic prescribing [85].

Physicians tap into their peers’ knowledge within their social and professional networks and rely on their community of practice as important sources of information for prescribing practices, even more so than national guidelines [86, 87]. Using social networks analyses, administrators can better identify well-connected practices (i.e., key opinion leaders) and target dissemination of best practices through key opinion leaders. In practice, when administrators are introducing new practice guidelines or implementing decision-support, gaining the confidence and support from well-connected physician practices within a local network may increase their use and spread.

Lastly, social capital has long been recognized as integral in individual and team performance. Previous research shows the association between social capital and the maturity of a hospital’s quality management system [88] and validated measurement tools for social capital within hospital management has subsequently been created [89]. Our research demonstrates the feasibility of using network metrics to monitor dimensions of individual social capital in relation to their embedded networks, and its association with quality outcomes such as prescribing. This provides a low-cost, highly replicable option that can be applied and scaled in a variety of settings due to the widely available administrative data in healthcare.

### Limitations

Our study is subject to limitations. First, we derived provider relationships based on the presence of patients shared within a year using claims data from one insurance company in each region, which may have overlooked certain links based on shared patients insured by other companies. We minimized the impact of this risk by using data from health insurance companies that have substantial market share in both regions within our study. Additionally, methods have advanced in recent years to restrict linkages among provider dyads to only those that share patients with one another over one episode of care [90]. While we do not have the data to consider this approach, this clinically intuitive method of identifying information-sharing relationships should be considered in future research.

Second, networks are subject to boundary specification problems. In this study, the boundaries are artificially drawn based on providers sought by residents within the two geographical areas. We assume that healthcare utilization in these two areas is relatively self-contained, which is conceivable as one area is a rural community with a natural boundary and the other is a relatively deprived neighborhood in a larger city. Further considering that patients tend to seek care in their communities that are culturally appropriate, we assume in our study that those living in Billstedt-Horn district typically seek care within the district. Third, while there is no standard gate-keeping system in Germany, general practitioners remain well-positioned to coordinate care for patients. Where more comprehensive data with wider geographic coverage is available, future research should consider a general practitioner-only network to better investigate the relationship between prescribing quality and peer relationships. Fourth, pharmaceutical influence on prescribing is widely substantiated with a study showing 84% of physicians indicating weekly pharmaceutical sales representatives visits in Germany [91]. It is conceivable that physician practices with a higher percentage of senior patients may be targetted more frequently by pharmaceutical sales representatives which inadvertently contributes towards to prescribing quality as higher prescribing volume has previously been associated with poor prescribing quality [92]. Fifth, where multi-year data is available, future research may account for provider-level fixed effects such as practice style, preference, and experience, thus increasing the precision of the estimate. Lastly, like most network research, this study was cross-sectional and thus hinders our ability to determine causality. While our objective was to characterize the association between centrality measures and prescribing performance among practices, it is likely that poorly performing practices have been operating for longer, and hence are further away from updated best practices and prescribe based on experience. Established practices are likely to have an established network of peers and accumulate more patients within the community, which drives up the measured centrality indicators. Future studies with richer data sources may consider longitudinal designs to determine the directionality of this association.

## Conclusions

Our study builds on the momentum of research in patient-sharing network analysis in the last decade and finds well-connected practices to be associated with increased over- and under-prescribing for senior patients in two regions in Germany. From this, we conclude that practices occupying strategic positions at the edge of networks with advantageous access to novel information are associated with better prescribing outcomes, whereas highly connected practices embedded in insulated information environments are associated with poor prescribing performance.

### Supplementary Information

**Additional file 1.**

## Data Availability

The data associated with the paper are not publicly available due to legal restrictions imposed by the health insurance companies providing the data (data contains potentially identifying patient information). The data set supporting the conclusions of this article are owned by German statutory health insurance and are subject to strict data protection rules according to the German social security code. Therefore, the data cannot be made publicly accessible. Data access for this research project was granted to the data processing organization (OptiMedis AG) based on a bilateral contract with the statutory health insurance companies. The data we accessed is collected by the health insurance fund when health providers bill their services. To fulfill the legal requirements to obtain the data, data users must agree on a contract with the statutory health insurer regarding data access. The licensee is permitted to use the data for the purpose as set out in the contract. Licensees are not allowed to pass the data to a third party. For assistance in obtaining access to the data, please contact the corresponding author (SYW).

## Abbreviations

BH: Billstedt Horn
FORTA: Fit fOR The Aged
PIM: Potentially inappropriate medications
WMK: Werra Meissner Kreis
