# Supplementary material for: Can peer effects explain prescribing appropriateness? a social network analysis
Source: BMC Med Res Methodol. 2023 Oct 28;23:252. doi: 10.1186/s12874-023-02048-7 (PMC10613382; doi:10.1186/s12874-023-02048-7)
Supplement: Supplementary file 1 — Additional file 1. [file 12874_2023_2048_MOESM1_ESM.docx]

**Supplement**

**S1** – Details on patient-sharing network mapping

We first create a bipartite (2-mode) network consisting of both patients and physicians, linked through patient visits during the calendar year. The bipartite network was then projected onto a unipartite network whereby remaining nodes represent physicians, and the linkages represent patients shared between the nodes (**Figure 1**). We eliminated ties between nodes that consisted of fewer than 8 shared patients in the same calendar year due to previous findings that ties identified by fewer than 8 shared patients have a higher chance of being spurious links driven by patient choice.


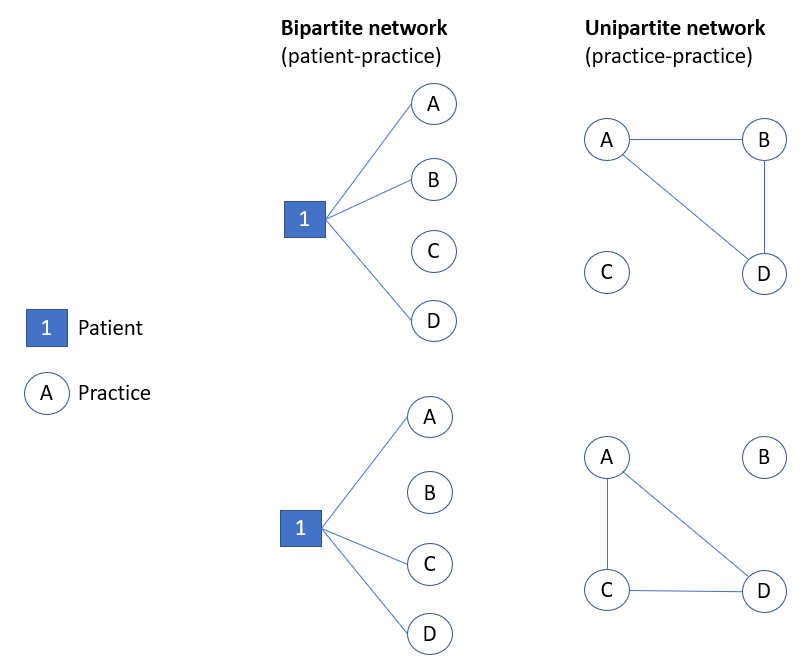


**Figure 1. Construction of patient-sharing network.** Bipartite network can be constructed where patients and practices are connected via clinical encounters identified through claims data. This bipartite network can be reduced to a unipartite network consisting only of practices through the patient that is shared between them.

To create the bipartite affiliation network, we first created an edge list consisting of the practice and patient from each insurance claim, and then restructured it as an incidence matrix whereby an incidence of 1 is recorded if the practice and patient are associated based on the claims (***Figure 2***, following the codes provided in this tutorial: https://solomonmg.github.io/post/working-with-bipartite-affiliation-network-data-in-r/). The incidence matrix is then transposed onto itself to produce the adjacency matrix, which is then used to create the network graph using the igraph package in R. The adjacency matrix contains information on the interpersonal collaborative ties between the practices, with each row and column representing individual practices and the intersecting cells representing the intensity of the tie (*i.e.*, the number of shared patients between the two practices.)
